# Evidence against a temporal association between cerebrovascular disease and Alzheimer’s disease imaging biomarkers

**DOI:** 10.1038/s41467-023-38878-8

**Published:** 2023-05-29

**Authors:** Petrice M. Cogswell, Emily S. Lundt, Terry M. Therneau, Carly T. Mester, Heather J. Wiste, Jonathan Graff-Radford, Christopher G. Schwarz, Matthew L. Senjem, Jeffrey L. Gunter, Robert I. Reid, Scott A. Przybelski, David S. Knopman, Prashanthi Vemuri, Ronald C. Petersen, Clifford R. Jack

**Affiliations:** 1 Department of Radiology, Mayo Clinic, 200 First St SW, Rochester, MN 55905 USA; 2 Department of Quantitative Health Sciences, Mayo Clinic, 200 First St SW, Rochester, MN 55905 USA; 3 Department of Neurology, Mayo Clinic, 200 First St SW, Rochester, MN 55905 USA; 4 Department of Information Technology, Mayo Clinic, 200 First St SW, Rochester, MN 55905 USA

**Keywords:** Biomarkers, Alzheimer's disease, Cerebrovascular disorders

## Abstract

Whether a relationship exists between cerebrovascular disease and Alzheimer’s disease has been a source of controversy. Evaluation of the temporal progression of imaging biomarkers of these disease processes may inform mechanistic associations. We investigate the relationship of disease trajectories of cerebrovascular disease (white matter hyperintensity, WMH, and fractional anisotropy, FA) and Alzheimer’s disease (amyloid and tau PET) biomarkers in 2406 Mayo Clinic Study of Aging and Mayo Alzheimer’s Disease Research Center participants using accelerated failure time models. The model assumes a common pattern of progression for each biomarker that is shifted earlier or later in time for each individual and represented by a per participant age adjustment. An individual’s amyloid and tau PET adjustments show very weak temporal association with WMH and FA adjustments (R = −0.07 to 0.07); early/late amyloid or tau timing explains <1% of the variation in WMH and FA adjustment. Earlier onset of amyloid is associated with earlier onset of tau (R = 0.57, R^2^ = 32%). These findings support a strong mechanistic relationship between amyloid and tau aggregation, but not between WMH or FA and amyloid or tau PET.

## Abstract (web summary)

Whether a relationship exists between cerebrovascular disease and Alzheimer’s disease has been a source of controversy. Here, the authors show there is a very weak temporal relationship between the progression of Alzheimer’s disease biomarkers and those of cerebrovascular disease.

## Introduction

Alzheimer’s disease (AD) pathologic changes (e.g. amyloid and tau aggregation), and cerebrovascular disease (CVD) often coexist, and there is a growing body of literature supporting that pathologic changes and imaging features of CVD contribute to cognitive decline^1–6^. In vivo assessment of AD-related changes of amyloid and tau aggregation is most reliably performed with amyloid and tau PET^7^. White matter hyperintensities (WMH) and fractional anisotropy (FA), an indicator of loss of microstructural integrity, are imaging features commonly thought to arise from small vessel disease (SVD)-related changes and are considered biomarkers of CVD^8,9^. However, recent literature has suggested that these white matter changes could be mechanistically linked with AD pathologic changes. Pathology, ex vivo imaging-pathology, and in vivo imaging studies have shown topographic associations between biomarkers of AD and CVD. Parietal WMH has been proposed to be related primarily to degeneration secondary to amyloid and tau deposition based on pathology studies of AD and non-AD participants^10,11^. Pathology and imaging-pathology correlation studies have shown an association between arteriosclerosis or WMH and cortical tau aggregation, particularly in posterior regions of the aging population^12–14^. In vivo imaging studies have shown an association between amyloid, but not tau, PET SUVR, and WMH^15–19^. WMH has also been implicated as a feature of AD based on its association with autosomal dominant AD mutation carriers^20^.

However, the coexistence of pathology does not necessarily imply a causal relationship, and therefore, despite topographic association of AD and CVD, it remains unclear whether AD and CVD are mechanistically linked processes. Establishing temporal relationships between AD and CVD biomarker progression may provide additional insight into potential mechanistic associations. One method to assess the temporal evolution of a biomarker is an accelerated failure time (AFT) model, which assumes a common pattern of progression for a given biomarker that is shifted earlier or later in time for each individual^21^. Each individual may be assigned an adjustment for each biomarker of interest that represents how much earlier or later the individual is estimated to progress on that biomarker relative to the population mean. An association of these individual adjustments would indicate a temporal association of biomarker change; for example, if an individual progressed earlier on amyloid PET, would that individual also progress earlier on WMH?

In this work, we investigate the relationship of an individual’s estimated timing of progression of AD imaging biomarkers, amyloid and tau PET, and CVD imaging biomarkers, WMH and FA, via an AFT model. An individual’s amyloid and tau PET adjustments showed very weak temporal association with WMH and FA adjustments; the relative timing of an individual’s progression on amyloid or tau explained <1% of the variation in the timing of progression of WMH and FA. Whereas the onset of amyloid explained 32% of the variation in the onset of tau. These findings support a strong mechanistic relationship between amyloid and tau aggregation, but not between WMH or FA and amyloid or tau PET.

## Results

### Participants

The study included 2406 participants from the Mayo Clinic study of Aging (MCSA), a population-based observational study, or the Mayo Alzheimer’s Disease Research Center (ADRC), a clinic-based observational cohort. Participants had a mean (SD) age of 74 (11) years, 1161 (48%) were female, and 1817 (76%) were cognitively unimpaired (CU), 303 (13%) had mild cognitive impairment (MCI), and 286 (12%) had Alzheimer clinical syndrome dementia (AlzCS Dem) (Table 1). The emphasis of this work was not on behavior of the different clinically defined groups; the demographics of these groups were provided to highlight clinical characteristics. All participants had at least one MRI and one amyloid (PiB) PET scan. The number of MRI scans per participant ranged from 1 (950, 39% participants) to 12 with approximately 5% of participants with 5 or more MRI scans. Approximately half of the participants had one amyloid PET (1179/2406, 49%), 585 (24%) had two amyloid PET, and 643 (27%) had three or more amyloid PET scans. A total of 966/2406 (40%) of participants did not have a tau ([18 F]flortaucipir) PET scan, 867 (36%) had one, 380 (16%) had two, and 193 (8%) had three or more tau PET scans.

**Table. 1:** MCSA and ADRC participant and scan characteristics at the most recent visit

	CU (*N* = 1817)	MCI (*N* = 303)	AlzCS Dem (*N* = 286)	Total (*N* = 2406)
Age, years	73 (11)	80 (9)	73 (10)	74 (11)
Female sex	881 (48%)	132 (44%)	148 (52%)	1161 (48%)
Education, years	15 (3)	14 (3)	16 (3)	15 (3)
APOE ε4 genotype^a^				
Non-carrier	1268 (70%)	182 (60%)	76 (27%)	1526 (63%)
Carrier	457 (25%)	111 (37%)	172 (60%)	740 (31%)
Study				
ADRC	0 (0%)	0 (0%)	257 (90%)	257 (11%)
MCSA	1817 (100%)	303 (100%)	29 (10%)	2149 (89%)
No. total amyloid-PETs				
1	901 (50%)	150 (50%)	128 (45%)	1179 (49%)
2	453 (25%)	61 (20%)	71 (25%)	585 (24%)
3+	463 (25%)	92 (30%)	87 (30%)	642 (27%)
No. total tau-PETs				
0	727 (40%)	149 (49%)	90 (31%)	966 (40%)
1	683 (38%)	77 (25%)	107 (37%)	867 (36%)
2	304 (17%)	37 (12%)	39 (14%)	380 (16%)
3+	103 (6%)	40 (13%)	50 (17%)	193 (8%)
No. total FLAIR-MRIs				
0	8 (0%)	2 (1%)	13 (5%)	23 (1%)
1	732 (40%)	112 (37%)	90 (31%)	934 (39%)
2	495 (27%)	78 (26%)	64 (22%)	637 (26%)
3+	582 (32%)	111 (37%)	119 (42%)	812 (34%)
No. total DTI-MRIs				
0	222 (12%)	46 (15%)	132 (46%)	400 (17%)
1	754 (41%)	117 (39%)	92 (32%)	963 (40%)
2	502 (28%)	70 (23%)	37 (13%)	609 (25%)
3+	339 (19%)	70 (23%)	25 (9%)	434 (18%)
Amyloid-PET, SUVR	1.55 (0.36)	1.93 (0.58)	2.43 (0.46)	1.70 (0.50)
Tau-PET, SUVR	1.20 (0.10)	1.29 (0.21)	1.98 (0.54)	1.31 (0.35)
WMH %	0.88 (0.97)	1.50 (1.17)	1.38 (1.28)	1.01 (1.06)
FA GCC	0.62 (0.03)	0.60 (0.03)	0.59 (0.03)	0.61 (0.03)

^a^ Individuals having unknown APOE ε4 genotype were included in analyses (92 CU, 10 MCI, 38 AlzCS Dem), hence the sum of the count of carriers and non-carriers will not equal the column totals.

Counts are displayed as *n* (%) and numeric values as mean (SD).

### Model fits

An AFT model^21^, a nonlinear mixed effects model, was fit with amyloid (PiB) PET global meta-ROI SUVR, tau PET (flortaucipir) temporal meta-ROI SUVR, WMH as a percent of total intracranial volume (TIV), and FA in the genu of the corpus callosum (FA GCC) as the quad-variate endpoints, and sex, APOE ε4 genotype status, and years of education as covariates. The percent of WMH volume to TIV (WMH%) was used in analyses to account for differences based on head size. FA GCC was used as an indicator of white matter tract integrity and cerebrovascular injury based on prior work showing that this metric best captures variability in systemic vascular health^9^. The model output included (1) a per participant/endpoint random effect that represents an individual’s adjustment or time-shift for each biomarker and indicates if the participant’s biomarker progressed earlier or later relative to their demographic peers, accounting for the covariates and (2) the correlation (R) between adjustments for each pair of biomarkers.

**Fig. 1:**
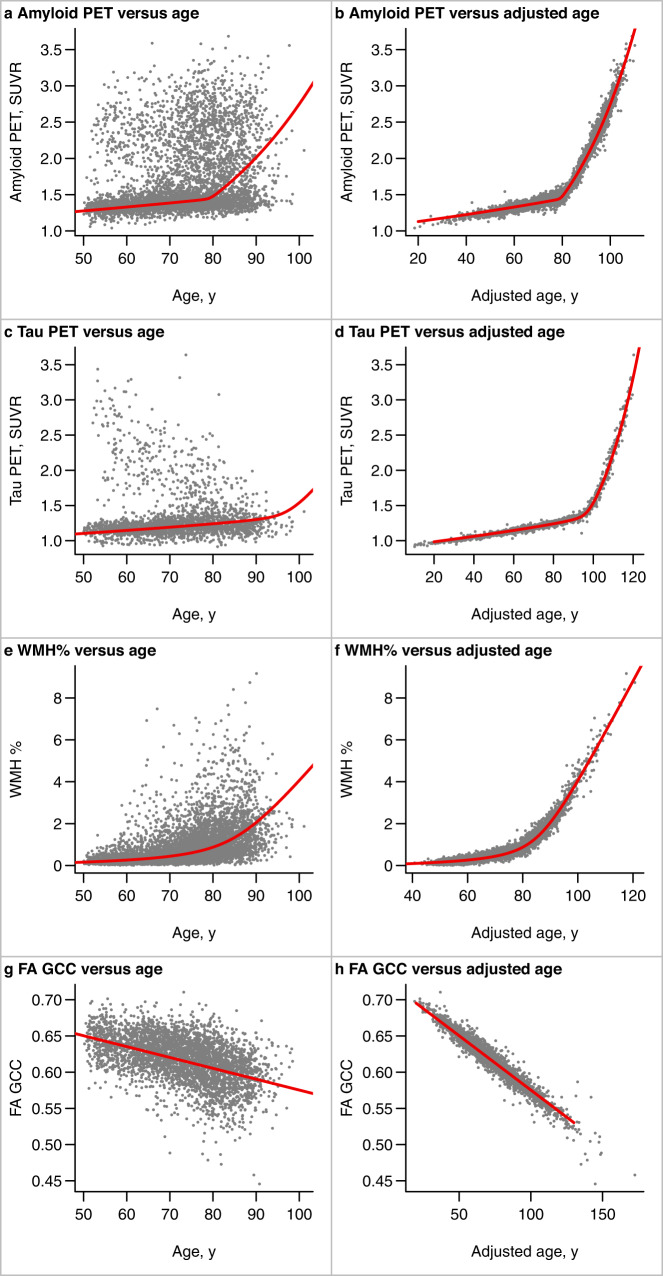
Relationships between age and PET or MRI outcomes from the primary AFT model fit in the MCSA + ADRC. Scatter plots of amyloid PET SUVR, tau PET SUVR, WMH%, and FA GCC vs age (**a**, **c**, **e**, **g**) and adjusted age (**b**, **d**, **f**, **h**). The adjusted age is the participant’s estimated age with respect to the biomarker of interest based on both the covariate and random effects. Each dot represents one observation, and individuals having serial data contribute multiple observations: amyloid PET (*n* = 4640), tau PET (*n* = 2249), WMH% (*n* = 5261), FA GCC (*n* = 3635). The red curves indicate a hypothetical common curve; we assume all individuals follow this trajectory of biomarker progression with the curve shifted left or right based on the random effects and covariates effects. Source data are provided as a Source Data file. *ADRC = Mayo Alzheimer’s Disease Research Center, AFT = accelerated failure time model, FA GCC = fractional anisotropy in the genu of the corpus callosum, MCSA = Mayo Clinic Study of Aging, SUVR = standardized uptake value, WMH% = white matter hyperintensity volume scaled as % of total intracranial volume.

The relationships between amyloid PET SUVR, tau PET SUVR, WMH%, and FA GCC with age and adjusted age are shown in Fig. 1. The x-axis in the left column (age) is the participant’s biological age while the x-axis in the right column (adjusted age) is the participant’s estimated age with respect to the biomarker of interest after accounting for both the covariate and random effects. The red curve indicates a hypothetical common curve; we assume all individuals follow this trajectory of biomarker progression with the curve shifted left or right based on the random effects and covariates effects. The model assumption that all participants follow the same trajectory for a given biomarker is supported by similar trajectories among the subset of participants with three or more measurements (Supplemental Fig. 1). The curves for amyloid PET SUVR, tau PET SUVR, and WMH had an inflection point beyond which the biomarker value rapidly increases. The association of FA GCC with age and adjusted age was linear.

### Covariate effects

The covariate effects (Table 2) for each measure report the estimated time-shift. For example, an estimate of 9.0 (7.8, 10.2) for APOE ε4 genotype on amyloid implies an amyloid accumulation that began, on average, 9.0 (7.8, 10.2) years earlier in APOE ε4 carriers than non-carriers. APOE ε4 carriers also had earlier tau accumulation on average (6.1 [3.9, 8.3] years); however, there was no evidence of an APOE ε4 effect on vascular progression. The largest covariate effect was referral to the ADRC on tau PET of 29.4 (27.1, 31.8) years earlier.

**Table. 2:** Covariate effects for the primary model fit in the MCSA + ADRC

	Amyloid PET	Tau PET	WMH%	FA GCC
APOE ε4 carrier	9.0 (7.8, 10.2)	6.1 (3.9, 8.3)	−0.9 (−2.0, 0.2)	−0.5 (−2.1, 1.2)
Female sex	2.1 (1.1, 3.1)	2.6 (0.8, 4.5)	2.3 (1.4, 3.3)	4.1 (2.7, 5.6)
Education 1-yr	0.2 (0.0, 0.4)	0.8 (0.4, 1.1)	−0.1 (−0.3, 0.1)	−0.1 (−0.4, 0.1)
Referral to ADRC	19.0 (18.0, 20.0)	29.4 (27.1, 31.8)	10.4 (9.3, 11.4)	13.3 (11.2, 15.4)

Values shown are mean (95% credible interval). Estimates are in years and represent estimated adjustment or years by which that biomarkers progression is shifted earlier (positive) or later (negative) with vs without that covariate.

### Association of individual-level adjustments

**Fig. 2:**
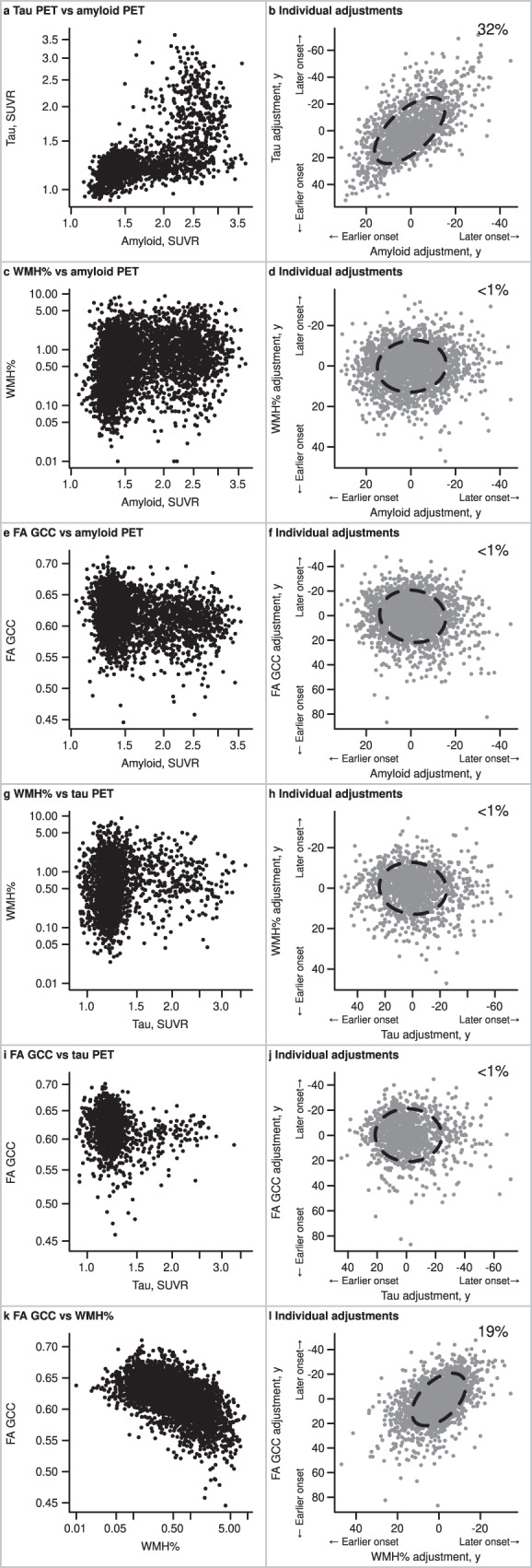
Relationships of biomarker values and individual-level adjustments in the MCSA + ADRC. In the left column (panels **a**, **c**, **e**, **g**, **i**, **k**), scatter plots display each 2-way relationship between the outcome measures used in the model; each dot represents one observation, and a participant could have multiple observations. The number of observations varies across comparisons based on data availability: tau PET SUVR with amyloid PET SUVR (*n* = 2243), WMH% with amyloid PET SUVR (*n* = 4587), FA GCC with amyloid PET SUVR (*n* = 3388), WMH% with tau PET SUVR (*n* = 2196), FA GCC with tau PET SUVR (*n* = 1321), and FA GCC with WMH% (*n* = 3626). In the right column (panels **b**, **d**, **f**, **h**, **j**, **l**), scatter plots summarize each 2-way relationship between the model output of individual adjustments. The individual adjustments are shown in years and indicate whether a participant’s level of disease burden was consistent with earlier onset or later onset relative to their demographic peers; each dot represents one participant, and the number of participants varies across the comparisons based on data availability: tau PET SUVR with amyloid PET SUVR (*n* = 1440), WMH% with amyloid PET SUVR (*n* = 2383), FA GCC with amyloid PET SUVR (*n* = 2006), WMH% with tau PET SUVR (*n* = 1417), FA GCC with tau PET SUVR (*n* = 1119), and FA GCC with WMH% (*n* = 2004). An 80% ellipse indicates the strength of association between the y-axis variable onset adjustment for a given x-axis variable onset adjustment; a perfect circle would indicate no relationship between adjustments. The x-axes and y-axes are flipped for individual adjustments. A higher positive value or earlier onset relative to the population mean is shown to the left of the x-axis and bottom of the y-axis. The percent variation explained (square of the correlation*100) between individual-level adjustments is given in the upper right-hand corner. Source data are provided as a Source Data file. *ADRC = Mayo Alzheimer’s Disease Research Center, FA GCC = fractional anisotropy in the genu of the corpus callosum, MCSA = Mayo Clinic Study of Aging, SUVR = standardized uptake value, WMH% = white matter hyperintensity volume scaled as % of total intracranial volume.

The association between the individual-level adjustments or time-shifts as determined by the model are shown in Fig. 2 and Table 3. There was a strong association of the individual-level adjustments for amyloid and tau PET, R (95% credible interval, CI) = 0.57 (0.52, 0.61); variation in amyloid PET onset (early/late timing) explained approximately one-third (0.57^2^ = 32%) of the variation in tau PET onset. WMH% and FA GCC adjustments showed a modest association, R (95% CI) = 0.44 (0.39, 0.48). However, amyloid PET SUVR and WMH% adjustments showed essentially no association, R (95% CI) = 0.07 (0.02, 0.12); whether an individual progressed early or late on amyloid PET SUVR accounted for <1% (0.07^2^ = 0.5%) of WMH% onset. The association of amyloid PET SUVR with FA GCC, tau PET SUVR with WMH%, and tau PET SUVR with FA GCC adjustments were similarly very weak (R^2^ = < 1% for each).

**Table. 3:** Correlation coefficient, R (95% credible interval), between individual-level adjustments for the primary model fit in the MCSA + ADRC

	Tau PET	WMH %	FA GCC
Amyloid PET	0.57 (0.52, 0.61)	0.07 (0.02, 0.12)	−0.07 (−0.12, −0.01)
Tau PET		−0.07 (−0.13, −0.004)	−0.01 (−0.08, 0.06)
WMH%			0.44 (0.39, 0.48)

### Sensitivity analysis – parietal WMH%

When parietal WMH%, the region most highly implicated in association with AD in prior literature^2^, was used in the model in place of global WMH%, the results were similar (Supplemental Table 1). Specifically, the association between individual-level adjustments of amyloid and tau PET SUVR with parietal WMH% remained very weak, R^2^ < 1%.

### Sensitivity analysis – tau PET Braak regions

When we repeated modeling using tau PET Braak stages 1–2, 3–4, and 5–6 in place of the temporal meta-ROI, to evaluate for differential temporal associations with CVD biomarkers based on tau PET distribution in early vs late disease stages, the results remained unchanged. The individual adjustments of the three Braak stages were very strongly associated (R = 0.82–0.98) (Supplemental Fig. 2). The association of each of the tau PET Braak stages individual adjustments with amyloid PET, WMH%, and FA GCC and covariate effects closely followed that of the primary analyses (Supplemental Tables 2 and 3). The associations of the amyloid PET and tau PET Braak stage individual adjustments ranged from 0.53 (0.49, 0.57) for Braak 1-2 to 0.44 (0.41, 0.48) for Braak 5-6. The APOE ε4 carrier effect ranged from 4.9 (3.7, 6.1) for Braak 1-2 to 1.6 (0.4, 3.0) for Braak 5-6.

### Secondary analysis – associations with neurodegeneration

**Fig. 3:**
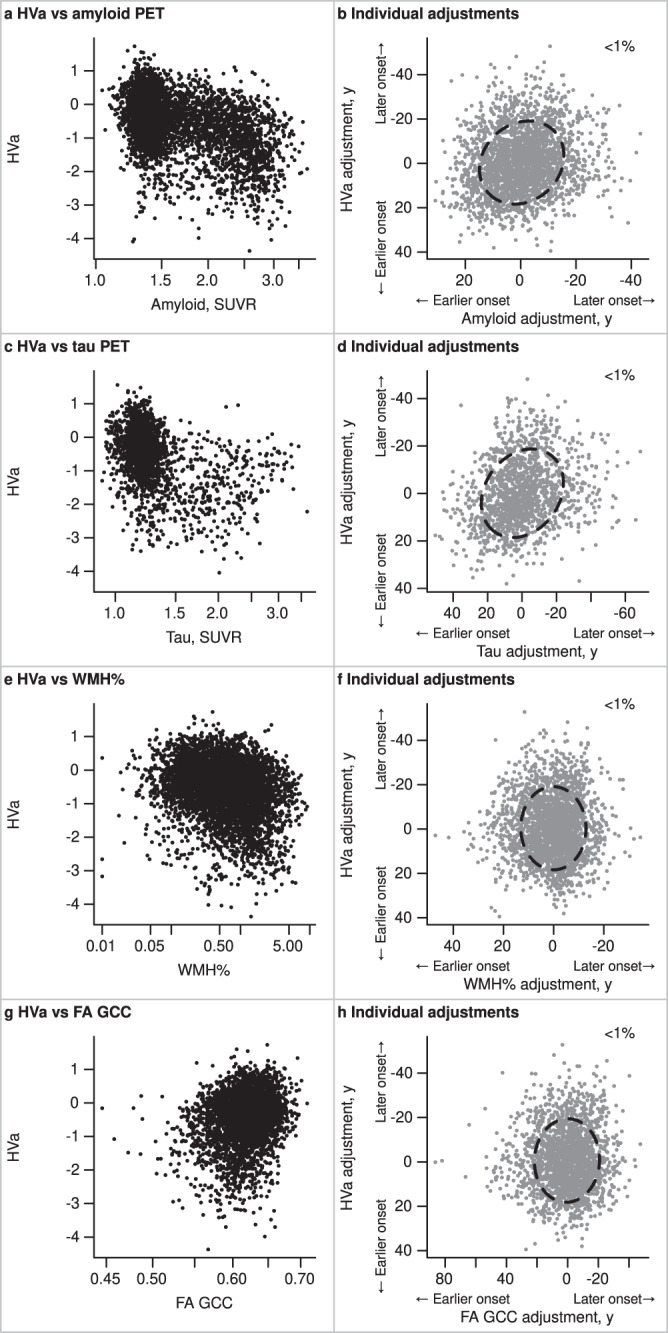
Relationships of AD (amyloid and tau PET) and CVD (WMH% and FA GCC) biomarker values and individual adjustments with those of hippocampal volume (HVa) in the MCSA + ADRC. In the left column (panels **a**, **c**, **e**, **g**), scatter plots display each 2-way relationship between the outcome measures used in the model; each dot represents one observation, and a participant could have multiple observations. The number of observations varies across comparisons based on data availability: HVa with amyloid PET SUVR (*n* = 4640), HVa with tau PET SUVR (*n* = 2249), HVa with WMH% (*n* = 5261), and HVa with FA GCC (*n* = 3635). In the right column (panels **b**, **d**, **f**, **h**), scatter plots summarize each 2-way relationship between the model output of individual adjustments. The individual adjustments are shown in years and indicate whether a participant’s level of disease burden was consistent with earlier onset or later onset relative to their demographic peers; each dot represents one participant, and the number of participants varies across the comparisons based on data availability: HVa with amyloid PET SUVR (*n* = 2406), HVa with tau PET SUVR (*n* = 1440), HVa with WMH% (*n* = 2383), and HVa with FA GCC (*n* = 2006). An 80% ellipse indicates the strength of association between the y-axis variable onset adjustment for a given x-axis variable onset adjustment; a perfect circle would indicate no relationship between adjustments. The x-axes and y-axes are flipped for the individual adjustments. A higher positive value or earlier onset relative to the population mean is shown to the left of the x-axis and bottom of the y-axis. The percent variation explained (square of the correlation × 100) between individual-level adjustments is given in the upper right-hand corner. Source data are provided as a Source Data file. *ADRC = Mayo Alzheimer’s Disease Research Center, FA GCC = fractional anisotropy in the genu of the corpus callosum, HVa = hippocampal volume adjusted for head size, MCSA = Mayo Clinic Study of Aging, SUVR = standardized uptake value, WMH% = white matter hyperintensity volume scaled as % of total intracranial volume.

To complete the investigation of the AT(N) triad, we extended the primary model to include hippocampal volume adjusted for head size (HVa) as a measure of neurodegeneration. The covariate effects followed similar trends as for amyloid and tau PET, with the largest effect being referral to the ADRC of 19.4 (18.5, 20.3), followed by APOE ε4 carriership of 3.6 (2.2, 4.9) years earlier (Supplemental Table 4). The association of the HVa with tau PET and amyloid PET individual adjustments were R (95% CI) = 0.22 (0.16, 0.27) and 0.16 (0.11, 0.21), respectively (Fig. 3 and Supplemental Table 5). There was essentially no association between HVa and WMH% or FA GCC individual adjustments, (R^2^ < 0.1%).

### Validation in ADNI

**Fig. 4:**
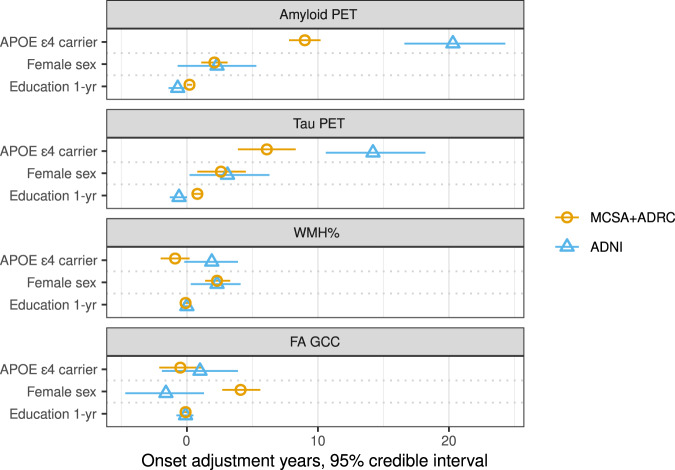
Covariate effects for each outcome by independent cohort. Values shown are mean (95% credible interval). Estimates are in years and represent estimated adjustment or years by which that biomarkers progression is shifted earlier (positive) or later (negative) with vs without that covariate. A model was fit on each of cohorts: MCSA + ADRC shown in orange circles and ADNI shown in blue triangles. In statistical analysis, each participant was an independent observation with *n* = 2406 for the MCSA + ADRC and n = 740 for ADNI. Source data are provided as a Source Data file. * ADNI = Alzheimer’s Disease Neuroimaging Initiative, ADRC = Mayo Alzheimer’s Disease Research Center, FA GCC = fractional anisotropy in the genu of the corpus callosum, MCSA = Mayo Clinic Study of Aging, SUVR = standardized uptake value, WMH% = white matter hyperintensity volume scaled as % of total intracranial volume.

**Fig. 5:**
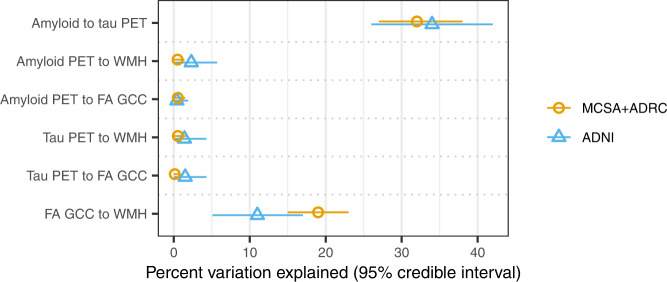
Percent variance explained of individual adjustments for pairs of outcomes, with estimates shown by cohort. Values shown are R^2^ with 95% credible intervals. Estimates computed from models fit on each of two cohorts: MCSA + ADRC shown in the orange circle and ADNI in a blue triangle. In the statistical analyses, each participant was a unique observation, and the number of participants varies across the comparisons based on data availability. For the MCSA + ADRC: amyloid PET SUVR with tau PET SUVR (*n* = 1440), amyloid PET SUVR with WMH% (*n* = 2383), amyloid PET SUVR with FA GCC (*n* = 2006), tau PET SUVR with WMH% (*n* = 1417), tau PET SUVR with FA GCC (*n* = 1119), and FA GCC with WMH% (*n* = 2004). For ADNI: amyloid PET SUVR with tau PET SUVR (*n* = 660), amyloid PET SUVR with WMH% (*n* = 723), amyloid PET SUVR with FA GCC (*n* = 572), tau PET SUVR with WMH% (*n* = 665), tau PET SUVR with FA GCC (*n* = 527), and FA GCC with WMH% (*n* = 575). Source data are provided as a Source Data file. * ADNI = Alzheimer’s Disease Neuroimaging Initiative, ADRC = Mayo Alzheimer’s Disease Research Center, FA GCC = fractional anisotropy in the genu of the corpus callosum, MCSA = Mayo Clinic Study of Aging, SUVR = standardized uptake value, WMH% = white matter hyperintensity volume scaled as % of total intracranial volume.

The Alzheimer’s Disease Neuroimaging Initiative (ADNI)−3 study was used as an independent validation cohort. The validation cohort included 740 participants with mean (SD) age of 74 (8) years, 384 (52%) were female, 438 (59%) were CU, 212 (29%) had MCI, and 90 (12%) had dementia (Supplemental Table 6). Covariate effects in ADNI showed similar trends as in the MCSA + ADRC (Fig. 4 and Supplemental Table 7). The largest covariate effects were APOE ε4 carriership on amyloid and tau PET, which were even greater than seen in the MCSA + ADRC. As in the MCSA + ADRC, the individual adjustments of amyloid and tau PET were strongly associated, R = 0.58 (0.51, 0.64); whether an individual progresses early/late on amyloid accounted for approximately 35% variability in the relative timing of tau accumulation (Fig. 5 and Supplemental Table 8). Timing of FA GCC progression accounted for ~10% variability timing of WMH change. Timing of amyloid and tau PET change account for <2% variability in timing of progression of WMH and FA GCC.

## Discussion

In this work, we used an AFT model to study the association of individual adjustments in timing of AD and CVD biomarker progression. These findings are important in informing the proposed relationships between AD (defined as an accumulation of pathological aggregated amyloid and tau proteins) and small vessel cerebrovascular disease. The individual adjustments of AD biomarkers (amyloid and tau PET) and established CVD biomarkers (WMH and FA GCC) showed weak to no association. In other words, whether an individual accumulates amyloid and tau earlier or later relative to the population provides no information regarding when they will develop WMH and loss of integrity of the white matter tracts in the genu of the corpus callosum. Therefore, although WMH and amyloid or tau may coexist in similar regions, the lack of a temporal relationship of when one vs the other progresses on an individual level suggests that these processes occur by independent mechanisms. The distinction of these mechanistic processes is important for the development of targeted therapies. Therapies targeted at reducing cardiovascular risk factors may decrease CVD-related imaging changes (WMH, FA) and associated cognitive decline. However, altering the course of CVD would not be anticipated to directly affect AD-related changes of amyloid and tau accumulation and subsequent neurodegeneration and cognitive decline.

These findings were validated in an independent cohort (ADNI). In both the MCSA + ADRC and ADNI cohorts, early/late amyloid explained about 35% of whether tau was early/late, WMH% and FA GCC 10-19% of each other’s timing, and interactions of amyloid and tau PET with WMH% and FA GCC, 2% or less. Minor differences in the individual adjustments between cohorts may be related to the differences in data acquisition and processing techniques, as well as greater technical variability in the ADNI data secondary to the combination of data acquired at many different sites and scanners. The covariate effects showed a similar trend between cohorts. The larger APOE ε4 carriership effect on amyloid and tau PET in ADNI compared to the MCSA + ADRC may be related to a higher fraction of APOE ε4 carriers in the ADRC than MCSA and some of the APOE ε4 effect being ascribed to study difference (referral to the ADRC). The wider credible intervals in ADNI, for both the covariate effects and correlations of individual adjustment, are likely a result of both a smaller sample size used in the analysis and technical variation. Overall, the replication of our findings in a separate cohort supports the validity and generalizability of the model.

The sensitivity analyses support the main findings. When restricting WMH to the parietal region, the region where WMH has been shown to have the highest association with amyloid and tau PET as well as cognitive decline^2,11,13^, the relationship of WMH and amyloid PET individual adjustments remained very weak.

When using tau PET Braak stages 1–2, 3–4, and 5–6 in place of the tau PET temporal meta-ROI, the individual adjustments of each of the Braak stages with amyloid PET, WMH%, and FA GCC were similar to one another and to the results of the tau PET meta-ROI. These findings indicate temporal progression of imaging changes related to CVD are unrelated to the timing of tau accumulation throughout the early to late stages of tau accumulation on PET. However, when a person progressed on amyloid was closely related to the estimated relative timing of tau accumulation in each of the Braak stages. Finally, the strength of the association of the individual adjustments of amyloid (as well as APOE ε4 carriership) with the Braak stages follow the expected trend based on the temporal ordering of tau accumulation in the Braak stages, R (amyloid PET, Braak 1-2) > R (amyloid PET, Braak 3-4 > R (amyloid PET, Braak 5-6), which provides further internal validation of the model.

Other recent studies similarly refute the hypothesis of mechanistic associations of AD and CVD. A twins study showed no genetic or environmental associations between amyloid burden and CVD, as measured by WMH volume and mean diffusivity^22^. In the Atherosclerosis Risk in Communities (ARIC) study, hypertension and amyloid accumulation (amyloid PET) were independent predictors of dementia risk^23^. Findings in an Asian memory clinic cohort study and meta-analysis support that amyloid deposition and CVD or WMH are independent, additive processes that contribute to cognitive decline^24,25^. Finally, a recent cross-sectional imaging study showed that diffusion metrics are associated with age and WMH but not amyloid and tau^26^.

The adjustments or time-shifts of AD biomarkers, amyloid and tau PET, were highly associated, as seen in similar work^21^. These findings support a strong mechanistic relationship of amyloid and tau, as anticipated based on prior modeling of AD biomarker progression and in keeping with the amyloid cascade hypothesis^27–29^. Additionally, demonstrating the expected association of amyloid and tau PET provides validation for the model fits and hypothetical trajectories of biomarker progression used in this work.

Changes in WMH% and FA were modestly associated, in keeping with prior work showing that these metrics both result from SVD-related changes^9,30–32^. The lower association of the individual adjustments of WMH% and FA compared to amyloid and tau PET may reflect a less direct association of FA with WMH compared to that of amyloid and tau. This is supported by a recent study by Shen et al., which showed that age and cardiovascular risk factors may contribute directly to WMH or indirectly through changes in FA^30^. Additionally, the temporal trajectories of WMH% and FA were different. WMH%, amyloid PET SUVR, and tau PET SUVR model fit showed a slow increase at younger ages followed by a dramatic rise beyond the inflection point, which suggests a tipping point, such as disruption in the balance of protein production and clearance for both amyloid and tau. However, the FA data fit showed a linear, step-wise change, as has been demonstrated in prior work^33^. The difference in temporal trajectories of WMH and FA suggests that these biomarkers capture different facets of SVD-related changes.

The primary goal of this work was to evaluate potential mechanistic relationships between AD and CVD biomarkers. Although neurodegeneration is not specific to either of these processes, hippocampal volume was added to the MCSA + ADRC model to complete the assessment of the AT(N) triad and further validate the model performance. As expected, none of the AD or CVD biomarkers showed strong associations with the progression of hippocampal volume loss. Tau PET showed the strongest, though still a relatively weak, association; the timing of tau PET progression accounted for ~5% of the timing of progression of HVa. These findings support that tau accumulation is more proximal to neurodegeneration than amyloid^27^ and that while AD pathology leads to hippocampal volume loss, there are non-AD processes that also result in neurodegeneration^34^. Although some prior cross-sectional studies have found associations between WMH and hippocampal volume^35^, we did not detect an association between the timing of the progression of these processes. This may indicate intermediary processes or that these pathology progress primarily along independent pathways.

The model identified an ADRC referral effect, as has been seen in prior studies including both population-based and clinical referral samples^21^. Those individuals referred for enrollment in the ADRC on average progressed earlier on amyloid and tau PET compared to those in the MCSA, which in part is due to the presence of individuals with early-onset AD in the ADRC. The ADRC sample also progressed earlier on WMH and FA GCC. These findings of progressing early on amyloid and tau and to a lesser degree on WMH and FA does not tell us anything about individual associations. Rather, individuals referred for clinical care of dementia may have either AD and CVD changes, or both, that are contributing to their cognitive decline. APOE ε4 carriership was also associated with a positive amyloid and tau adjustment or earlier age of onset, as has been seen in other cohorts and modeling techniques^36^.

There are limitations to this study. The model assumes all individuals follow the same trajectory for each biomarker, which is a generalization that allows for the mapping of biomarker change over a longer time period than captured in a single participant. A similar single trajectory with time shifts has been found in other studies of amyloid PET^36,37^. The primary analyses evaluated the timing of the progression of biomarkers within representative regions, as these are the regions most closely associated with the pathology of interest^9,38^. Findings were similar with the use of the parietal WMH%, which has been of particular interest in the study of AD-CVD biomarker associations, and tau PET Braak stages, which allowed targeted evaluation of associations in early vs later disease stages.

In conclusion, we found an association between individual adjustments or timing of progression between amyloid and tau PET, and between WMH% and FA GCC. However, the adjustments of AD biomarkers (amyloid and tau PET) showed essentially no association with the adjustments of CVD biomarkers (WMH and FA GCC). The lack of temporal association of AD vs CVD biomarkers suggests that they occur via independent mechanisms.

## Methods

### Participants

The study included participants in the Mayo Clinic Study of Aging (MCSA), a longitudinal cohort study of individuals residing in Olmsted County, Minnesota, or in the Mayo Alzheimer’s Disease Research Center (ADRC), a longitudinal study of patients enrolled through the clinical practice. For inclusion in the study, all participants were required to have complete demographic information of sex and education, have at least one PET (amyloid or tau) and one MRI performed after 2009, and be age 50 years or older at the time of PET scan. MCSA participants were required to have a diagnosis of cognitively unimpaired (CU), mild cognitive impairment (MCI), or Alzheimer’s clinical syndrome dementia (AlzCS Dem) at their most recent visit, and ADRC participants a diagnosis of AlzCS Dem. The clinical diagnosis was determined by an expert panel utilizing established criteria and medical history, neurologic examination, and detailed neuropsychological exam^39–41^. Participants with these diagnoses were selected for inclusion as we were interested in studying AD biomarker progression across the clinical spectrum.

### Standard protocol approvals, registrations, and patient consents

The study was approved by the Mayo Clinic and Olmsted Medical Center institutional review boards. All participants provided informed written consent; consent was obtained from a legally authorized representative for cognitively impaired participants as necessary.

### MR imaging

Imaging was performed on 3 T GE (GE Healthcare, Waukesha, WI) or Siemens (Siemens, Erlangen, Germany) systems; MR imaging was performed on the GE system prior to and through March of 2018 and on the Siemens system starting October of 2017 and after. The MRI sequences included 3D T1-weighted imaging with Magnetization Prepared Rapid Acquisition Gradient Recalled Echo (MPRAGE), T2-weighted fluid attenuated inversion recovery (FLAIR), and diffusion tensor imaging (DTI). The GE acquisition parameters were: MPRAGE: TR/TE 2300/3.0 ms, TI 900 ms, flip angle 8°, FOV 260 × 260 mm, matrix 256 × 256, phase FOV 94%, slice thickness 1.2 mm; axial 2D T2*-*weighted FLAIR: TR/TE 11000/147 ms, TI 2250 ms, flip angle 90°, FOV 220 × 220 mm, matrix 256 × 192, slice thickness 3 mm. DTI was performed using an axial spin-echo echo planar imaging (EPI) sequence with 2.7  mm^3^ isotropic resolution, five b = 0 followed by 41 b = 1000 s/m^2^ diffusion-weighted volumes. The Siemens acquisition parameters were: MPRAGE: TR/TE 2300/3.1 ms, TI 945 ms, flip angle 9°, FOV 240 × 256 mm, matrix 320 × 300, slice thickness 0.8 mm; 3D T2-weighted FLAIR: TR/TE 4800/441 ms, TI 1550 ms, flip angle 120°, FOV 256 × 256 mm, matrix 256 × 256, slice thickness 1.2 mm. DTI was performed using Simultaneous Multi-Slice (SMS) acceleration with adaptive coil combination^42^, TR/TE 3400/71 ms, FOV 232 × 232 mm, matrix 116 × 116, slice thickness 2 mm, 13 b = 0 followed by 6 b = 500, 48 b = 1000, and 60 b = 2000 s/mm^2^ diffusion-weighted images.

### White matter hyperintensity (WMH) volume

WMH volume was estimated using the MPRAGE and T2-weighted FLAIR sequences via a fully automated algorithm, updated from a previously described in-house semi-automated method^15,43^. In brief, WMH were identified and segmented based on location and intensity relative to gray matter and neighboring white matter voxels. False positives were removed by applying a white matter mask derived from the SPM12^44^ segmentation of MPRAGE, and removing single isolated voxel detections. The percent of WMH to TIV (as derived from SPM segmentation) was used in analyses to account for differences based on head size and is expressed as WMH%. Global and lobar WMH% were calculated using the Mayo Clinic Adult Lifespan Template (MCALT) Lobar atlas (https://www.nitrc.org/projects/mcalt/)^45^.

### DTI analysis

The DTI data were preprocessed using previously described methods that include Gibbs ringing correction, skull stripping, denoising, debiasing, and distortion correction^9,26^. Diffusion tensors were fit, and fractional anisotropy (FA) values were calculated. Regional median FA was computed using an in-house version of the John Hopkins University Eve white matter atlas^46^. The FA in the genu of the corpus callosum (FA GCC) was used as an indicator of white matter tract integrity and cerebrovascular injury based on prior work showing that this metric is most closely associated with vascular disease-related changes^9,47–49^. Of note, although FA in all white matter tracts has been found to be highly correlated, certain regions have shown higher associations with specific disease processes. In this work, we used FA as an indicator of cerebrovascular injury and therefore chose to interrogate the GCC. Other regions may show a higher association with amyloid or tau accumulation and evaluating the temporal relationship of those changes with PET is beyond the scope of the current work^50,51^.

### Correcting for MR scanner manufacturer and protocol differences

A sample of 111 participants aged 34 to 97 (median 70 years), 57% male, and 66% cognitively unimpaired were imaged on both GE and Siemens within 3 days^52^. This sample was used to perform a Deming regression between GE and Siemens WMH% values to account for differences in scanner manufacturer and protocol. The regression equation for WMH % was: Siemens WMH% = −0.0357 + 0.9276 * GE WMH%. To avoid extrapolation, very small or negative mapped WMH% was set to 0.01, where 0.01 was the minimum observed WMH% from the Siemens scans. We used the same process for FA GCC. The regression equation for FA GCC derived from a subset of 81 participants with usable FA values was: Siemens FA GCC = 0.2644 + 0.5988 * GE FA GCC.

### PET imaging

Amyloid PET was performed with Pittsburgh compound B (PiB)^53^ on GE scanners (models Discovery 690XT, Discovery RX, and Discovery MI) or Siemens scanners (Biograph Vision 600). Four five-minute frames were acquired after a 40-minute uptake period, averaged, and processed using in-house pipelines^54,55^. The amyloid PET meta-ROI was derived via the voxel number weighted average of the median uptake in each of the prefrontal, orbitofrontal, parietal, temporal, anterior and posterior cingulate, and precuneus regions normalized to the cerebellar crus gray matter. Although some studies have shown regional staging or progression of amyloid PET^56–58^, evaluation of our population-based data and other cohorts suggests little to no meaningful regional variation^59–62^. Therefore, the global amyloid PET meta-ROI was used in these analyses.

Tau PET was performed with [18 F]flortaucipir (Avid Radiopharmaceuticals) on GE (models Discovery 690XT and Discovery MI) or Siemens scanners (Biograph Vision 600). Four five-minute frames were acquired after an 80-minute uptake period, averaged, and processed using a standard in-house pipeline^63^. The tau PET temporal meta-ROI was derived via the voxel number weighted average of the median uptake in each of the amygdala, fusiform, middle/inferior temporal, entorhinal, and parahippocampal regions normalized by the cerebellar crus gray matter. All available amyloid and tau PET scans for each participant were used in analyses. We have previously shown that PET SUVR measurements from these pipelines can be directly combined across MRI manufacturers without any adjustment^64^. Harmonization across PET scanners was performed by adjusting blurring parameters during the recon, following the method of Joshi et al.^65^.

### Modeling

An accelerated failure time (AFT) model^21^, a joint nonlinear mixed effects model, was implemented. This model fits all available relevant data from each individual in the cohort (single time point cross-sectional data from some participants and longitudinal data from others) to estimate individual-level adjustments (right-left shifts) of a hypothetical common curve^27,28^ indicating if a participant starts the accumulation process of a biomarker earlier or later than the population average. In the present study, we extended this prior AFT model to include four biomarkers: amyloid PET meta-ROI, tau PET temporal meta-ROI, global WMH%, and FA GCC. Amyloid and tau PET SUVR were natural log-transformed to account for skewness. FA GCC was modeled as 1 − FA such that an increase in value was indicative of progression for all biomarkers. The model includes a smooth progression function for each marker, per marker regression coefficients for APOE ε4 carriership, sex, education, and ADRC referral effects, and per participant age adjustments for each outcome.

Of particular interest are the estimated correlations of these adjustments, and the model output includes a correlation matrix of the random effects or the correlation of adjustments between pairs of biomarkers. This correlation will be denoted as R. Models were fit using Hamiltonian Markov Chain Monte Carlo (MCMC) using the rstan package version 2.21.8, R version 4.1.2. The code for the fits is available upon request. As described in a prior publication and supplement^66^, the model is set up so that each participant/measurement pair is a separate response/ line of data. This allows the fit to deal with different numbers of amyloid, tau, etc values for a given participant. The random effect for a missing biomarker measurement is treated as a free parameter in the MCMC, i.e., a random draw from the prior distribution. Other than some possible increase in iteration time, this has no adverse effect on the computation; such values are omitted from summaries and plots.

The per-participant age effects indicate how early or late each participant’s accumulation processes, relative to the population as a whole, proceed for amyloid PET, tau PET, WMH% and FA GCC. The covariate effects and adjustments are reported in years, e.g., a 4-year adjustment for amyloid implies that an individual is 4 years “older” with respect to the amyloid process, or equivalently, starts the accumulation process 4 years earlier. A larger positive shift or older adjusted age in the AFT model is equivalent to a longer duration in “amyloid chronicity” in the longitudinal model described by Koscik et al.^36,37^. All FA GCC results have been post-processed to restore native scaling such that a positive adjustment similarly indicates earlier onset or a decrease in brain connectivity.

### Sensitivity analysis – parietal WMH%

As imaging associations of WMH and amyloid PET have been found primarily in the parietal lobes in some prior work^2^, we performed a sensitivity analysis using parietal WMH% in place of global WMH%. We did not separately evaluate periventricular vs deep to subcortical WMH or additional lobar WMH regions as they have been shown to be highly associated in the MCSA/ADRC as well as other populations^67^.

### Sensitivity analysis – Braak tau PET stages

We repeated the modeling with tau PET Braak stages in place of the temporal meta-ROI to evaluate if associations between AD and CVD biomarkers differed with early vs late-stage topographic distributions of tau PET. We used three tau PET Braak stages: 1-2 (entorhinal cortex), 3-4 (voxel number weighted average of the following regions: parahippocampal, fusiform, lingual, amygdala, insula, inferior temporal, superior temporal pole, middle temporal pole, middle temporal, posterior cingulate, retrosplenial cortex, anterior cingulate, and mid cingulate), and 5-6 (voxel number weighted average of the following regions: inferior parietal, angular, middle orbitofrontal, inferior orbitofrontal, superior orbitofrontal, medial orbitofrontal, rectus, olfactory, superior temporal, Heschl, inferior frontal operculum, inferior frontal triangularis, supramarginal, superior occipital, middle occipital, inferior occipital, precuneus, superior parietal, superior frontal, superior medial frontal, supplemental motor area, middle frontal, paracentral lobule, postcentral, precentral, calcarine, cuneus, and rolandic operculum). Therefore, a total of six biomarkers (3 tau PET Braak stages, amyloid PET, global WMH%, and FA GCC) were used in the model.

### Secondary analysis - neurodegeneration

To further validate our findings, we extended the primary model to include hippocampal volume as a measure of neurodegeneration. The hippocampal volume (HV) was derived from SPM12 segmentation of the MPRAGE/T1-weighted MRI and adjusted for head size, denoted as adjusted hippocampal volume (HVa)^68^. Our T1-weighted MRI segmentation pipeline has been extensively validated and previously published^69^. Briefly, T1-weighted MRI were segmented and corrected for intensity inhomogeneity using Unified Segmentation from SPM12^44^, with tissue priors and settings from MCALT^45^. MCALT ADIR122 atlas regions were propagated from MCALT space to each image using Advanced Normalization Tools (ANTs) Symmetric Normalization^70^. The complete segmentation software is available from https://www.nitrc.org/projects/mcalt/. HVa was calculated as the residual from a linear regression of HV (y) versus TIV (x), using separate models for males and females. The equations were as follows: HVa (males) = observed HV – (7.58 + 0.00441*(observed TIV − 1500)) and HVa (females) = observed HV – (7.88 + 0.00476*(observed TIV − 1500))^71^.

### Validation cohort

ADNI was used as a validation cohort. We limited inclusion to ADNI-3, given the differences in MRI imaging protocols between ADNI-GO/2 and ADNI-3 (acquisition (http://adni-info.org). Other inclusion criteria were similar to those applied in the MCSA/ADRC cohort, with a few modifications based on study differences. All participants were required to have complete demographic information of sex, education, and APOE ε4 genotype, have at least one PET (amyloid or tau) and one MRI, be age 55 years or older at the time of PET scan, and have a diagnosis of CU, MCI, or dementia with etiology of Alzheimer’s disease only for MCI and dementia participants, based on the most recent visit. We obtained the following imaging metrics from LONI (https://www.loni.usc.edu/): amyloid PET centiloid^72,73^, temporal tau PET SUVR^54,74^, WMH volume^75^, and FA in the GCC^76^. The TIV was calculated in-house via SPM segmentation. The model was fit on: amyloid centiloid divided by 200, log transformation of tau SUVR, WMH% of TIV divided by 5, and FA scaled as 1-FA. Note, amyloid PET was performed with different tracers in ADNI vs the MCSA/ADRC (florbetapir or florbetaben vs PiB), the global meta-ROI was referenced to the whole cerebellum in ADNI vs. the cerebellar crus gray in the MCSA/ADRC, and the ADNI amyloid PET was analyzed using the centiloid vs SUVR values in the MCSA/ADRC. The tau PET meta-ROI and MRI metrics used in ADNI are comparable to those in the MCSA/ADRC.

### Reporting summary

Further information on research design is available in the Nature Portfolio Reporting Summary linked to this article.

## Supplementary information

Supplementary Material

Reporting Summary

## Data Availability

The MRI, PET, and other data from the Mayo Clinic Study of Aging and the Alzheimer’s Disease Research Center are available to academic and industry researchers under restricted access per study and IRB data sharing policies. Access can be obtained by submitting a request to the MCSA and ADRC Executive Committee (https://www.mayo.edu/research/centers-programs/alzheimers-disease-research-center/research-activities/mayo-clinic-study-aging/for-researchers/data-sharing-resources). The imaging and demographic data from ADNI-3 used in this study are available in the LONI database (https://www.loni.usc.edu/). Source data are provided with this paper.

## Source data

Source Data

## Code availability

The code is available at github.com/Therneau/AFTmodel.
