# Supplementary material for: Evidence against a temporal association between cerebrovascular disease and Alzheimer’s disease imaging biomarkers
Source: Nat Commun. 2023 May 29;14:3097. doi: 10.1038/s41467-023-38878-8 (PMC10226977; doi:10.1038/s41467-023-38878-8)
Supplement: Supplementary file 1 — Supplementary Material [file 41467_2023_38878_MOESM1_ESM.pdf]

Evidence against a temporal association between cerebrovascular disease and Alzheimer's disease imaging biomarkers

Supplementary Information

Supplementary Table 1: Correlation coefficient, R, and 95% credible interval (CI) between individual-level adjustments for the model fit in the MCSA+ADRC with parietal WMH% of TIV in place of global WMH%.

	Tau PET	Parietal WMH %	FA GCC
Amyloid PET	0.57 (0.52, 0.61)	0.09 (0.04, 0.14)	-0.06 (-0.12, -0.01)
Tau PET		-0.03 (-0.10, 0.03)	-0.00 (-0.07, 0.07)
Parietal WMH%			0.41 (0.36, 0.46)

Supplementary Table 2: Covariate effects for the model using three tau PET Braak stages in place of the tau PET temporal meta-ROI in the MCSA+ADRC. Values shown are mean (95% credible interval). Estimates are in years and represent estimated adjustment or years by which that biomarkers progression is shifted earlier (positive) or later (negative) with vs without that covariate.

	Amyloid PET	Braak 1-2	Braak 3-4	Braak 5-6	WMH%	FA GCC
APOE e4 carrier	8.5 (7.7, 9.4)	4.9 (3.7, 6.1)	2.6 (1.4, 3.9)	1.6 (0.4, 3.0)	-0.1 (-1.0, 0.8)	-0.2 (-1.2, 0.8)
Female sex	2.0 (0.9, 2.9)	2.1 (1.1, 3.1)	2.5 (1.5, 3.3)	2.9 (1.9, 3.8)	2.3 (1.4, 3.4)	3.3 (2.3, 4.3)
Education 1-yr	0.3 (0.1, 0.5)	0.7 (0.4, 0.9)	0.7 (0.5, 0.9)	0.7 (0.5, 0.9)	-0.1 (-0.2, 0.1)	-0.0 (-0.2, 0.2)
Referral to ADRC	17.1 (16.2, 18.0)	20.4 (19.1, 21.7)	21.0 (19.7, 22.2)	20.8 (19.5, 22.1)	9.2 (8.3, 10.2)	11.0 (9.6, 12.3)

Supplementary Table 3: Correlation coefficient, R (95% credible interval), between individual-level adjustments in the model using three tau PET Braak stages in place of the tau PET temporal meta-ROI in the MCSA+ADRC.

	Braak 1-2	Braak 3-4	Braak 5-6	WMH%	FA GCC
Amyloid	0.53 (0.49, 0.57)	0.48 (0.44, 0.51)	0.44 (0.41, 0.48)	0.08 (0.03, 0.12)	-0.00 (-0.05, 0.04)
Braak 1-2		0.90 (0.88, 0.91)	0.82 (0.81, 0.84)	-0.04 (-0.08, 0.02)	0.08 (0.03, 0.13)
Braak 3-4			0.98 (0.97, 0.98)	-0.01 (-0.06, 0.03)	0.11 (0.07, 0.15)
Braak 5-6				0.03 (-0.02, 0.07)	0.15 (0.11, 0.19)
WMH%					0.41 (0.37, 0.45)

Supplementary Table 4: Covariate effects for the model fit in the MCSA+ADRC with adjusted hippocampal volume (HVa) in addition to the primary model endpoints.

	Amyloid PET	Tau PET	WMH%	FA GCC	HVa
APOE e4 carrier	8.7 (7.6, 9.8)	5.7 (3.6, 7.8)	-1.1 (-2.1, -0.1)	-0.6 (-2.2, 1.0)	3.6 (2.2, 4.9)
Female sex	2.2 (1.2, 3.1)	2.5 (0.8, 4.3)	2.4 (1.4, 3.4)	4.1 (2.6, 5.7)	1.4 (0.2, 2.7)
Education 1-yr	0.2 (0.0, 0.4)	0.8 (0.4, 1.2)	-0.1 (-0.3, 0.1)	-0.2 (-0.5, 0.1)	0.3 (0.1, 0.6)
Referral to ADRC	20.0 (19.3, 20.7)	28.7 (26.8, 30.6)	9.2 (8.4, 10.0)	11.5 (9.8, 13.3)	19.4 (18.5, 20.3)

Supplementary Table 5: Correlation coefficient, R (95% credible interval), between individual-level adjustments in for the model fit in the MCSA+ADRC with adjusted hippocampal volume (HVa) in addition to the primary model endpoints.

	Tau PET	WMH %	FA GCC	HVa
Amyloid PET	0.57 (0.52, 0.61)	0.07 (0.01, 0.12)	-0.07 (-0.12, -0.01)	0.16 (0.11, 0.21)
Tau PET		-0.07 (-0.13, -0.01)	-0.00 (-0.07, 0.07)	0.22 (0.16, 0.27)
WMH%			0.44 (0.39, 0.48)	-0.01 (-0.06, 0.04)
FA GCC				0.01 (-0.04, 0.06)

Supplementary Table 6: Participant and scan characteristics of the ADNI validation cohort. Counts are displayed as n (%) and numeric values as mean (SD).

	CN (N=438)	MCI (N=212)	Dementia (N=90)	Total (N=740)
Age, years	73 (7)	75 (8)	77 (9)	74 (8)
Female Sex	259 (59%)	86 (41%)	39 (43%)	384 (52%)
Education, years	17 (2)	16 (3)	16 (2)	16 (2)
APOE e4 genotype				
Carrier	144 (33%)	90 (42%)	56 (62%)	290 (39%)
Non-carrier	294 (67%)	122 (58%)	34 (38%)	450 (61%)
No. total amyloid-PETs				
0	4 (1%)	4 (2%)	3 (3%)	11 (1%)
1	201 (46%)	123 (58%)	63 (70%)	387 (52%)
2	178 (41%)	76 (36%)	24 (27%)	278 (38%)
3+	55 (13%)	9 (4%)	0 (0%)	64 (9%)
No. total tau-PETs				
0	39 (9%)	18 (8%)	12 (13%)	69 (9%)
1	243 (55%)	122 (58%)	38 (42%)	403 (54%)
2	87 (20%)	45 (21%)	27 (30%)	159 (21%)
3+	69 (16%)	27 (13%)	13 (14%)	109 (15%)
No. total FLAIR-MRIs				
0	3 (1%)	3 (1%)	0 (0%)	6 (1%)
1	151 (34%)	58 (27%)	42 (47%)	251 (34%)
2	172 (39%)	59 (28%)	29 (32%)	260 (35%)
3+	112 (26%)	92 (43%)	19 (21%)	223 (30%)
No. total DTI-MRIs				
0	72 (16%)	63 (30%)	29 (32%)	164 (22%)
1	283 (65%)	81 (38%)	48 (53%)	412 (56%)
2	75 (17%)	62 (29%)	12 (13%)	149 (20%)
3+	8 (2%)	6 (3%)	1 (1%)	15 (2%)
Amyloid PET centiloid	24 (32)	42 (48)	87 (46)	37 (44)
Tau PET SUVR	1.2 (0.1)	1.3 (0.3)	1.6 (0.4)	1.3 (0.3)
WMH %	0.3 (0.5)	0.4 (0.8)	0.6 (0.9)	0.3 (0.7)
FA GCC	0.52 (0.05)	0.51 (0.05)	0.48 (0.05)	0.51 (0.05)

Supplementary Table 7: Covariate effects in ADNI validation cohort.

	Amyloid PET	Tau PET	WMH%	FA GCC
APOE e4 carrier	20.3 (16.6, 24.3)	14.2 (10.6, 18.2)	1.9 (-0.2, 3.9)	1.0 (-1.9, 3.9)
Female sex	2.3 (-0.7, 5.3)	3.1 (0.2, 6.3)	2.3 (0.3, 4.1)	-1.6 (-4.7, 1.3)
Education 1-yr	-0.7 (-1.4, -0.1)	-0.6 (-1.3, 0.0)	0.0 (-0.4, 0.4)	-0.1 (-0.8, 0.5)

Supplementary Table 8: Correlation coefficient, R (95% credible interval), between individual-level adjustments in ADNI validation cohort.

	Tau PET	WMH %	FA GCC
Amyloid PET	0.58 (0.51, 0.64)	0.14 (0.04, 0.24)	0.04 (-0.06, 0.14)
Tau PET		0.10 (0.00, 0.21)	0.11 (0.02, 0.21)
WMH%			0.32 (0.23, 0.42)

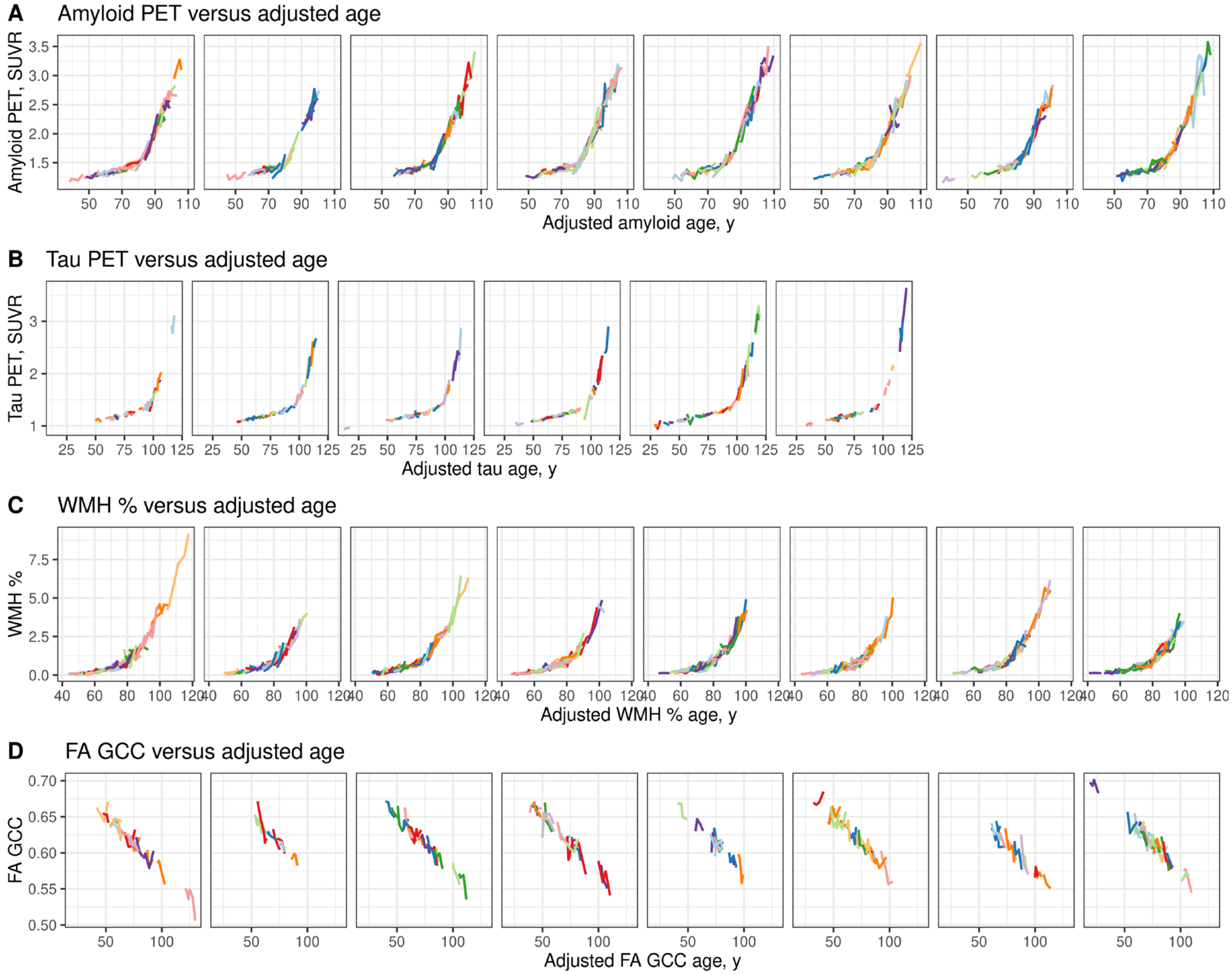

Supplementary Fig. 1: Trajectories of amyloid PET SUVR (A), tau PET SUVR (B), WMH% (C), and FA GCC (D) vs adjusted age for MCSA+ADRC participants with three or more longitudinal measures. For each biomarker, the assembled trajectories appear to fit the hypothetical common curve shown in **Fig. 1**. Each of the outcomes was split into multiple panels to minimize overlap and allow individual trajectories to be visualized. Source data are provided as a Source Data file.

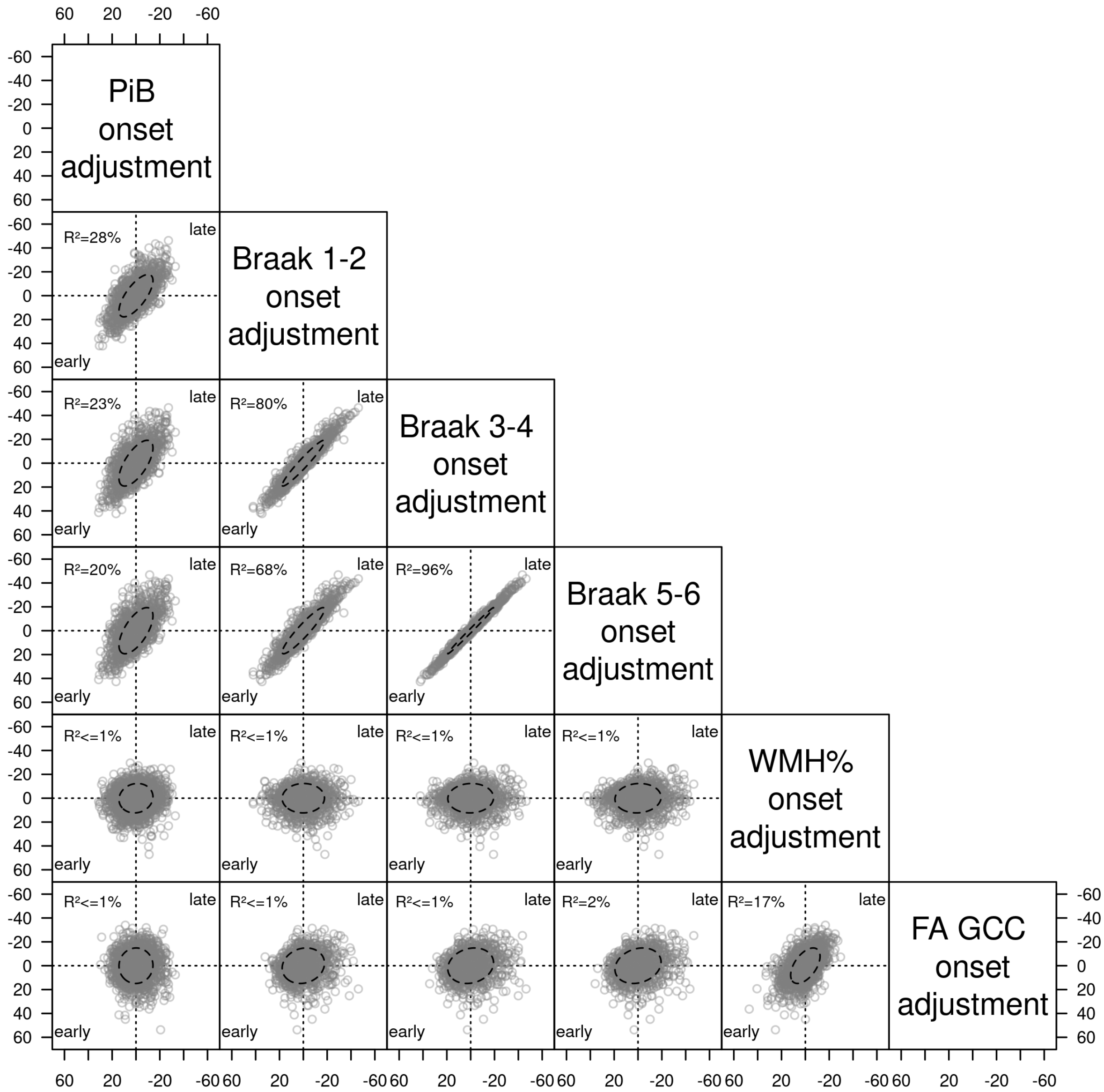

Supplementary Fig. 2: Relationships of individual-level adjustments between amyloid, three Braak tau PET stages, WMH%, and FA GCC. An 80% ellipse indicates the strength of association between the y-axis variable onset adjustment for a given x-axis variable onset adjustment; a perfect circle would indicate no relationship between adjustments. The percent variation explained (square of the correlation*100) between individual-level adjustments is given in the upper left-hand corner. Source data are provided as a Source Data file.
