# Supplementary material for: Evidence against a temporal association between cerebrovascular disease and Alzheimer’s disease imaging biomarkers
Source: Nat Commun. 2023 May 29;14:3097. doi: 10.1038/s41467-023-38878-8 (PMC10226977; doi:10.1038/s41467-023-38878-8)
Supplement: Supplementary file 2 — Reporting Summary [file 41467_2023_38878_MOESM2_ESM.pdf]

nature portfolio

Corresponding author(s): Petrice M. Cogswell

Last updated by author(s): 4/11/2023

Reporting Summary

Nature Portfolio wishes to improve the reproducibility of the work that we publish. This form provides structure for consistency and transparency in reporting. For further information on Nature Portfolio policies, see our Editorial Policies and the Editorial Policy Checklist.

Statistics

For all statistical analyses, confirm that the following items are present in the figure legend, table legend, main text, or Methods section.

n/a	Confirmed	
☐	☒	The exact sample size (*n*) for each experimental group/condition, given as a discrete number and unit of measurement
☐	☒	A statement on whether measurements were taken from distinct samples or whether the same sample was measured repeatedly
☒	☐	The statistical test(s) used AND whether they are one- or two-sided<br>*Only common tests should be described solely by name; describe more complex techniques in the Methods section.*
☐	☒	A description of all covariates tested
☐	☒	A description of any assumptions or corrections, such as tests of normality and adjustment for multiple comparisons
☐	☒	A full description of the statistical parameters including central tendency (e.g. means) or other basic estimates (e.g. regression coefficient) AND variation (e.g. standard deviation) or associated estimates of uncertainty (e.g. confidence intervals)
☒	☐	For null hypothesis testing, the test statistic (e.g. *F*, *t*, *r*) with confidence intervals, effect sizes, degrees of freedom and *P* value noted<br>*Give P values as exact values whenever suitable.*
☒	☐	For Bayesian analysis, information on the choice of priors and Markov chain Monte Carlo settings
☒	☐	For hierarchical and complex designs, identification of the appropriate level for tests and full reporting of outcomes
☒	☐	Estimates of effect sizes (e.g. Cohen's *d*, Pearson's *r*), indicating how they were calculated

Our web collection on statistics for biologists contains articles on many of the points above.

Software and code

Policy information about availability of computer code

Data collection	Medidata Rave was the software product used for data collection.
Data analysis	MRI processing was performed using SPM12 and ANTs. The Mayo Clinic Adult Lifespan Template (MCALT) Lobar atlas (https://www.nitrc.org/projects/mcalt/) and an in-house version of the John Hopkins Eve white matter atlas were applied for WMH and DTI analysis, respectively. Accelerated failure time models were fit using Hamiltonian Markov Chain Monte Carlo (MCMC) using the rstan package version 2.21.8, R version 4.1.2. Code for the fits is available at github.com/Therneau/AFTmodel.

For manuscripts utilizing custom algorithms or software that are central to the research but not yet described in published literature, software must be made available to editors and reviewers. We strongly encourage code deposition in a community repository (e.g. GitHub). See the Nature Portfolio guidelines for submitting code & software for further information.

Data

Policy information about availability of data

All manuscripts must include a data availability statement. This statement should provide the following information, where applicable:

- Accession codes, unique identifiers, or web links for publicly available datasets
- A description of any restrictions on data availability
- For clinical datasets or third party data, please ensure that the statement adheres to our policy

The MRI, PET, and other data from the Mayo Clinic Study of Aging and the Alzheimer's Disease Research Center are available to qualified academic and industry researchers under restricted access per study and IRB data sharing policies. Access can be obtained by submitting a request to the MCSA and ADRC Executive

Committee (https://www.mayo.edu/research/centers-programs/alzheimers-disease-research-center/research-activities/mayo-clinic-study-aging/for-researchers/data-sharing-resources).

Human research participants

Policy information about studies involving human research participants and Sex and Gender in Research.

Reporting on sex and gender	Sex is listed in the demographics table and included as a covariate in our model. Neither sex nor gender specific analyses were performed in this study.
Population characteristics	Mayo Clinic Study of Aging (MCSA): an age and sex stratified random sample of all residents of Olmsted County, Minnesota, over the age of 50 years, median age (Q1, Q3) = 75 (66, 82).<br>Mayo Alzheimer's Disease Research Center (ADRC): a longitudinal study of patients recruited from the clinical practice with median age (Q1,Q3) = 72 (64, 78) years.
Recruitment	The study included participants in the MCSA, a longitudinal cohort study of individuals residing in Olmsted County, Minnesota, or in the ADRC, a longitudinal study of patients enrolled through the clinical practice. The ADRC includes patients with early-onset AD whom on average progress earlier on imaging biomarkers. This effect is captured by including an ADRC referral effect as a covariate in the models.
Ethics oversight	The study was approved by the Mayo Clinic and Olmsted Medical Center institutional review boards. All participants provided informed written consent; consent was obtained from a legally authorized representative for cognitively impaired participants as necessary.

Note that full information on the approval of the study protocol must also be provided in the manuscript.

Field-specific reporting

Please select the one below that is the best fit for your research. If you are not sure, read the appropriate sections before making your selection.

☒ Life sciences ☐ Behavioural & social sciences ☐ Ecological, evolutionary & environmental sciences

For a reference copy of the document with all sections, see nature.com/documents/nr-reporting-summary-flat.pdf

Life sciences study design

All studies must disclose on these points even when the disclosure is negative.

Sample size	Sample size calculation was not performed. Data from all participants meeting inclusion criteria was included in the study. Sample size was determined to be adequate based on the parameters of the model fits.
Data exclusions	We employed strict inclusion criteria. "For inclusion in the study, all participants were required to have complete demographic information of sex and education, have at least one PET (PiB or Tau) and one MRI performed after 2009, and be age 50 years or older at the time of PET scan. MCSA participants were required to have a diagnosis of cognitively unimpaired (CU), mild cognitive impairment (MCI) or Alzheimer clinical syndrome dementia (AlzCS Dem) at their most recent visit and ADRC participants a diagnosis of AlzCS Dem." No participants meeting these criteria were excluded.
Replication	The findings of the primary model were independently replicated in ADNI-3.
Randomization	Data was not divided into experimental groups. All data was included in a single model and adjusted for covariates of APOE genotypes, sex, education, and age.
Blinding	All imaging metrics used in this study were generated by automated processing pipelines independent of patient diagnosis or other imaging metrics.

Reporting for specific materials, systems and methods

We require information from authors about some types of materials, experimental systems and methods used in many studies. Here, indicate whether each material, system or method listed is relevant to your study. If you are not sure if a list item applies to your research, read the appropriate section before selecting a response.

Materials & experimental systems

n/a	Involved in the study
☒	☐ Antibodies
☒	☐ Eukaryotic cell lines
☒	☐ Palaeontology and archaeology
☒	☐ Animals and other organisms
☐	☒ Clinical data
☒	☐ Dual use research of concern

Methods

n/a	Involved in the study
☒	☐ ChIP-seq
☒	☐ Flow cytometry
☐	☒ MRI-based neuroimaging

Clinical data

Policy information about clinical studies

All manuscripts should comply with the ICMJEguidelines for publication of clinical research and a completedCONSORT checklist must be included with all submissions.

Clinical trial registration	None
Study protocol	Not publicably available, as the studies are not clinical trials.
Data collection	Data was collected at Mayo Clinic Rochester. Imaging was performed 2009-2022.
Outcomes	The primary endpoint was the correlation of individual-level adjustments and the secondary endpoints the adjusted age, as determined via an accelerated failure time model.

Magnetic resonance imaging

Experimental design

Design type	NA (Functional MR imaging data was not used in this study.)
Design specifications	NA
Behavioral performance measures	NA

Acquisition

Imaging type(s)	Structural and diffusion
Field strength	3 Tesla
Sequence & imaging parameters	The MRI sequences included 3D Magnetization Prepared Rapid Acquisition Gradient Recalled Echo (MPRAGE), T2-weighted fluid attenuated inversion recovery (FLAIR), and diffusion tensor imaging (DTI). The GE acquisition parameters were: MPRAGE: TR/TE 2300/3.0 ms, TI 900 ms, flip angle 8º, FOV 260 × 260 mm2, matrix 256 × 256, phase FOV 94%, slice thickness 1.2 mm; axial 2D T2-weighted FLAIR: TR/TE 11000/147 ms, TI 2250 ms, flip angle 90º, FOV 220 × 220 mm2, matrix 256 × 192, slice thickness 3 mm. DTI was performed using an axial spin-echo echo planar imaging (EPI) sequence with 2.7 mm3 isotropic resolution, five b=0 followed by 41 b=1000 s/m2 diffusion-weighted volumes. The Siemens acquisition parameters were: MPRAGE: TR/TE 2300/3.1 ms, TI 945 ms, flip angle 9°, FOV 240 x 256 mm2, matrix 320 x 300, slice thickness 0.8 mm; 3D T2w FLAIR: TR/TE 4800/441 ms, TI 1550 ms, flip angle 120°, FOV 256 x 256 mm2, matrix 256 x 256, slice thickness 1.2 mm. DTI was performed using Simultaneous Multi-Slice (SMS) acceleration with adaptive coil combination (Caruyer et al., 2013), TR/TE 3400/71 ms, FOV 232 x 232 mm2, matrix 116 x 116, slice thickness 2 mm, 13 b=0 followed by 6 b=500, 48 b=1000, and 60 b=2000 s/mm2 diffusion-weighted images.
Area of acquisition	Whole brain

Diffusion MRI ☒ Used ☐ Not used

Parameters: The DTI was performed with a multishell acquisition and consisted of 127 volumes with 13 non-diffusion-weighted images (b=0 s/mm2), and 114 diffusion encoding gradient directions (6 b=500, 48 b=1000 and 60 b=2000 s/mm2).

Preprocessing

Preprocessing software	Segmentations were derived from statistical parametric mapping 12.
Normalization	Normalization to the MCALT template. Rigid registration between T2 FLAIR and MPRAGE sequences.
Normalization template	Mayo Clinic Adult Lifespan Template (MCALT) (Schwarz et al., 2017) space (https://www.nitrc.org/projects/mcalt)

Noise and artifact removal	The DTI data was preprocessed using previously described methods that include Gibbs ringing correction, skull stripping, denoising, debiasing, and distortion correction (Raghavan et al., 2021; Vemuri et al., 2018).
Volume censoring	Not applicable.

Statistical modeling & inference

Model type and settings	Joint nonlinear mixed effects models were used.
Effect(s) tested	Predicted time of biomarker change for an individual was the effect tested.

Specify type of analysis: ☐ Whole brain ☐ ROI-based ☒ Both

Anatomical location(s): Anatomic locations were based on probabilistic atlases.

Statistic type for inference (See Eklund et al. 2016)	Not applicable: Each of the four primary measures (amyloid and tau PET, WMH, FA) were based on pre-specified regions based on prior work and/or literature. No selection was performed.
Correction	Not applicable: The three covariates of APOE positivity, sex, and education were selected prior to analysis, based on existing literature.

Models & analysis

n/a	Involved in the study
☒	☐ Functional and/or effective connectivity
☒	☐ Graph analysis
☐	☒ Multivariate modeling or predictive analysis

Multivariate modeling and predictive analysis	Multivariate models included independent variables of sex, APOE, education, and referral to ADRC. The model had 8 chains totalling 20000 warm-up iterations, and 20000 post-warm-up iterations. We assessed convergence diagnostic R-hat and all were <=1.03. Sampling efficiency was measured using estimated effective sample size (bulk-ESS, tail-ESS). There were no divergent transitions after warm-up.
